# M6A associated TSUC7 inhibition contributed to Erlotinib resistance in lung adenocarcinoma through a notch signaling activation dependent way

**DOI:** 10.1186/s13046-021-02137-9

**Published:** 2021-10-16

**Authors:** Kai Li, Zi-Yang Peng, Shan Gao, Qing-Shi Wang, Rui Wang, Xiang Li, Guo-Dong Xiao, Jing Zhang, Hong Ren, Shou-Ching Tang, Xin Sun

**Affiliations:** 1 Department of Thoracic Surgery, the Second Department of Thoracic Surgery, Department of Thoracic Surgery and Oncology, Cancer Center, the First Affiliated Hospital of Xi’an Jiaotong University, 277 Yanta West Road, Xi’an City, 710061 Shaanxi Province China; 2 Department of Pathology, Anatomy & Cell Biology, Sidney Kimmel Cancer Center, Thomas Jefferson University, Philadelphia, PA 19107 USA; 3 Oncology Department, the First Affiliated Hospital of Zhengzhou University, Zheng Zhou City, 450052 Henan Province China; 4 University of Mississippi Medical Center, Cancer Center and Research Institute, 2500 North State Street, Jackson, MS 39216 USA

**Keywords:** Tyrosine kinase inhibitors, Notch signaling, Cancer stem cells, Therapy resistance, N6-methyladenosine

## Abstract

**Background:**

The small tyrosine kinase inhibitors (TKIs) subversively altered the lung cancer treatments, but patients will inevitably face the therapy resistance and disease recurrence. We aim to explore the potential roles of non-coding RNAs in sensitizing the TKIs effects. Methods: Multiple cellular and molecular detections were applied to confirm the mechanistic regulations and intracellular connections.

**Results:**

We explored the specific gene features of candidates in association with resistance, and found that m6A controlled the stemness of EMT features through METTL3 and YTHDF2. The miR-146a/Notch signaling was sustained highly activated in a m6A dependent manner, and the m6A regulator of YTHDF2 suppressed TUSC7, both of which contributed to the resistant features. Functionally, the sponge type of TUSC7 regulation of miR-146a inhibited Notch signaling functions, and affected the cancer progression and stem cells’ renewal in Erlotinib resistant PC9 cells (PC9ER) and Erlotinib resistant HCC827 cells (HCC827ER) cells. The Notch signaling functions manipulated the cMYC and DICER inner cytoplasm, and the absence of either cMYC or DICER1 lead to TUSC7 and miR-146a decreasing respectively, formed the closed circle to maintain the balance.

**Conclusion:**

PC9ER and HCC827ER cells harbored much more stem-like cells, and the resistance could be reversed by Notch signaling inactivation. The intrinsic miR-146 and TUSC7 levels are monitored by m6A effectors, the alternation of either miR-146 or TUSC7 expression could lead to the circling loop to sustain the new homeostasis. Further in clinics, the combined delivery of TKIs and Notch specific inhibitory non-coding RNAs will pave the way for yielding the susceptibility to targeted therapy in lung cancer.

**Supplementary Information:**

The online version contains supplementary material available at 10.1186/s13046-021-02137-9.

## Background

Lung cancer is the most dangerous cancer type worldwide, topping the cancer related mortality [1]. The lung cancer incidence tends to be stable, and even decreased slightly in western world [1, 2], but situations on cancer prevention were severe in developing China [3–5]. Exploring more sensitive screening strategy, improving the radical operation methods, or developing more effective adjuvant therapeutic agents was so urgent than ever [6, 7]. Lung adenocarcinoma consists of lots of therapy targets, and the EGFR Tyrosin Kinase related Inhibitors (TKIs) have been widely and effectively applied in clinical treatments for a decade, shortening the suffering process [8, 9]. However, inevitably, the targeted therapies must face the recurrence, and receive the second or third line of chemo-radiotherapy [10–12], and to identify more novel and effective therapeutic molecules and agents will be helpful and promising.

Traditional non-coding genes were always referred to the miRNAs when researching the post-translational regulations, and the lncRNAs (long non-coding RNAs) and circRNAs (circular RNAs) were later revealed for supplementing the affection of RNA family of non-coding members [13–17]. Individually, they could act as crucial modulator toward to certain downstream genes in many ways [18, 19]. The N-6-methyladenosine (m6A) regulation of RNAs attracted lots attention due to its specific and strong modification ability of epigenetic functions [20–22]. The m6A controller of writers, readers, and erasers could modulate the mRNA stability and translation, to mediate downstream effects [23, 24]. However, the roles of m6A in controlling the non-coding RNAs biogenesis were still not clear. To further identify the candidates to improve the TKIs treatments sensitization, we tentatively explored the supporting role of TUSC7 in cancer suppression, trying to establish the m6A correlated lncRNA functions in modulating the TKIs therapies resistance.

## Materials and methods

### Cell lines and cell culture

The human lung adenocarcinoma cells lines (PC9 and HCC827), and human embryonic cell line (HEK-293 T) were purchased from ATCC (American Type Culture Collection, VA, USA) or the Cell Bank of the Chinese Academy of Sciences (Shanghai, China). The Erlotinib-resistant cell lines (PC9/ER and HCC827/ER) were established by chronic exposure to increasing concentrations of drugs. The ectopic expression of TUSC or miR-146 in cells were constructed and restored as pervious study elucidated [25]. All cell lines were cultured in DMEM medium supplemented with 10% FBS (Gibco), 1% penicillin and 1% streptomycin, and incubated at 37 °C in a humidified atmosphere with 5% CO_2_.

### Materials and agents

The Notch inhibitors were chosen as FLI-06 (inhibitor-1, ab120633, Abcam), and γ-Secretase inhibitor (inhibitor-2, ab146170, Abcam). Erlotinib was purchased from Sigma-Aldrich (SML156-50MG, Merck KGaA, Darmstadt, Germany).

### Quantitative real-time PCR and western blot

Total RNA was extracted from cells using Trizol, according to the manufacturer’s protocol, and reverse-transcribed into cDNA by using SYBR RT-PCR kit (Takara, JAPAN). Real-time quantitative PCR (RT-qPCR) was performed with SYBR Premix ExTaqTM II Kit (Takara, JAPAN). The sequences of the primers for PCR were synthesized by Sangon Company (Shanghai, China) and were listed in Supplemental Materials and Methods. The relative expression of mRNA and miRNA were calculated by using the formula:2^-ΔΔCΤ^. For western blot analysis, the total protein from cell extracts was harvested using RIPA buffer contained protease inhibitors. The protein extracts were fractionated by 10% SDS-PAGE, transferred onto a nitrocellulose membrane, and then incubated with primary antibodies at 4 °C overnights, followed by HRP-conjugated secondary antibody (1:5000, #7074, Cell Signaling Technology) and visualized by using ECL Blotting Detection Reagents (Merck Millipore). The primary antibodies were as follows: anti-Notch1 (1:500; Rabbit mAb, #3608, Cell Signaling Technology), anti-Notch2 (1:1000, ab8926, Abcam), anti-NCSTN (1:1000, ab189125, Abcam), anti-Vinculin (1:8000, #4650, Cell Signaling Technology), anti-NUMB (1:1500, #2761, Cell Signaling Technology), anti-EGFR (1:2000, #4267, Cell Signaling Technology), anti-DICER1 (1:500, #5362, Cell Signaling Technology), anti-CMYC (1:1500, (9E10): sc-40, Santa Cruz), anti- Snail (1:1000, ab31787, Abcam), anti-METTL3 (1:2000, ab240595, Abcam), anti-YTHDF2 (1:1000, EPR20318, ab220163, Abcam).

### Sphere formation assay

Single-cell suspensions (1000 cells per well) of different groups were plated on six-well ultralow attachment plates (Corning Incorporated) in serum-free DMEM/F12 Medium supplemented with 20 ng/mL EGF (Invitrogen, Carlsbad, CA), b-FGF (Invitrogen, Carlsbad, CA) and 4 μg/mL insulin (Sigma-Aldrich, St. Louis, MO). After 1 week culture, the spheres of>50 μm was quantified by using an inverted microscope.

### Dual luciferase report assay

The putative sequences or mutant sequences of miR-146a target sites for TUSC7–3’UTR was synthesized and cloned into the pGL3 reporter vector (Promega). These constructed reporters were named pGL3-TUSC7-WT, pGL3-TUSC7-MUT. For luciferase assay, the cells were seeded onto 24-well plates and co-transfected with 200 ng of pGL3-TUSC7/EGFR-WT or pGL3-TUSC7/EGFR -MUT, 20 ng of pRL-TK plasmid as normalization control, together with miR-146a-5p mimic or miR-146a-5p control (GenePharma, Shanghai, China). After 48 h of transfection, Luciferase assays were performed by using the Dual Luciferase Reporter Assay System (Promega, WI, USA).

### ALDEFLUOR assay and fluorescence-activated cell sorting (FACS)

Aldehyde dehydrogenase (ALDH) enzyme activity in lung cells was determined by ALDEFLUOR assay kit (Stem Cell Technologies, Grenoble, France) according to the manufacturer’s instructions. Briefly, 1 × 10^6^/ml cells mixed with Aldefluor® assay buffer containing 1.5μΜ bodipy-aminoacetaldehyde (BAAA, an ALDH substrate). Then, the cell/substrate mixture was incubated for 1 h at 37 °C. Diethylaminobenzaldehyde (DEAB), a specific ALDH1 enzyme inhibitor, was used as negative control. The ALDH + population was detected in the green fluorescence channel (520–540 nm) of FACSAria (Becton Dickinson). Stained cells analyzed and sorted by utilizing FACSDiva (BD Biosciences) and Flow-Jo software (Treestar, Ashland, OR). Nonviable cells were excluded using 1 μg propidium iodide (PI; Sigma Aldrich, Vienna, Austria).

### RNA-immunoprecipitation

The cells (5 × 10^6^)/ml were harvested and washed twice with ice-cold 1 × PBS buffer. Collected cell pellet was lysed for 15 min on ice by RIP buffer for 30 min, and pretreated with a 1:10 dilution in NT2 buffer. The cell lysate was further centrifuged at 15,000×g for 15 min at 4 °C, followed by treated with magnetic beads conjugated to human anti-ATG3 antibody (1:50) or the control IgG for 18 h at 4 °C and further washed twice with cold NT2 Buffer. Magnetic beads subsequently mixed with the diluted lysates (10 μl beads/ml lysate). The mixture was re-suspended in 100 μl NT2 Buffer containing 30 μg proteinase K to digest the protein. Co-purified RNA was extracted by the TRIzol reagent and used in subsequent qRT-PCR assay.

### M6A methylation quantification assay

The m6A methylation status of cells detected using the m6A RNA Methylation Quantification Kit (Epigentek, Cat#P-9005-113) according to the manufacturer’s instructions. In brief, 200 ng of total RNA was used as an input respectively. Then RNA samples were captured and detected by spectrophotometer (Bio Tek Instruments, Inc. US) at 450 nm. The level of m6A methylation was calculated according to the manufacturer’s instructions.

As for LC–MS/MS assay, mRNA was purified from the total RNA using via oligo dT magnetic beads. Then 200 ng mRNAs were incubated with 0.5 U nuclease P1 in reaction system at 42 °C for about 1 h. After that, mRNAs were incubated with 3 μL of 1 M NH4HCO3 and 1 μL of 1 U/μL alkaline phosphatase at 37 °C for 2 h. And mRNAs were diluted and filtered. After which, a C18 column were used to separate mRNAs [26–29]. Then mRNAs were analyzed by an Agilent (6410 QQQ) triple-quadrupole LC mass spectrometer. Calibration curves were used to calculate the Ratio of m6A to A.

### Nude mouse xenograft model

A total of 21 4-week-old female BALB/cA-nu nude mice were purchased from Beijing Huafukang Biosciences (Beijing, China), then we maintained them in specific pathogen-free conditions. Control vector, TUSC7 knockout, FLI-06 treated H1975 cells (1*10^7^) cells were suspended in 100 μL of serum-free DMEM medium (Hyclone, USA), mixed with matrix gel (Corning, USA), and then were injected subcutaneously. The changes in the tumor size were recorded every 3 or 5 days. We calculated the tumor volume using the following formula: V = 1/2 × l × w2 (l is the longer axis, 2 is the shorter axis). All mice were sacrificed 28 days after the injection of cells. The dissected tumor samples were immersed in 4% paraformaldehyde (BioSharp, China) and embedded in paraffin.

### Statistical analysis

Statistical analysis was carried out by using Graph Pad Prism 6 and SPSS 20.0 software (SPSS Inc., Chicago, IL, USA). All numerical data were expressed as mean ± standard deviation (SD). Experiments were carried out with three or more replicates. Two or more groups were assessed by using Student’s t test or ANOVA individually. *P* < 0.05 was considered to be statistically significant.

## Results

### Notch signaling activation in lung adenocarcinoma pointed to poor survival expectance

**Fig. 1:**
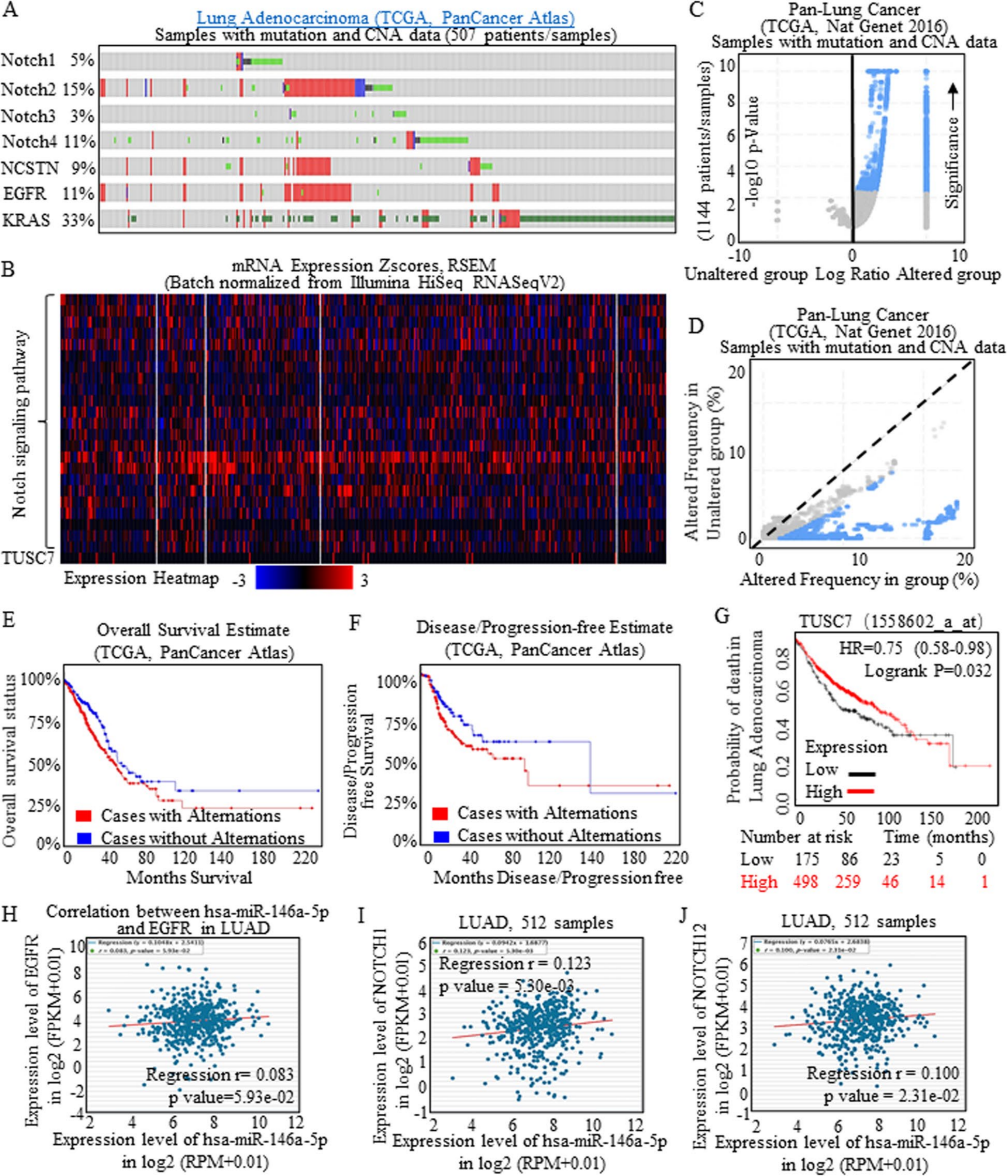
Aberrant Notch signaling activation in Lung adenocarcinoma. Notch signaling of NOTCH1, NOTCH2, NOTCH3, NOTCH4, DVL1, NUMB, NCSTN, APH1A, SNW1, DTX2, DTX3, DTX1, NCOR2, CTBP2, CTBP1, HDAC1, HDAC2, CIR1, RBPJ, RBPJL, CREBBP, KRAS, MAPK1, MAPK1 were applied for expression level detection, and a total of 507 lung adenocarcinoma samples with mutation and CNA data (TCGA, Pan-Cancer Atlas) were collected. **A** Expression plots showed the key members of Notch signaling were amplified, together with EGFR overexpression, and KRAS mutations. **B** Heat map results revealed the universal overexpression of Notch signaling participants, and the TUSC7 result was limited due to the mRNA expression screening system restrictions. Specifically, expression level of TUSC7 showed the reverse consistency with the Notch signaling activation patterns. The TCGA samples data with mutation and CAN from Pan-Lung Cancer (Nat Genet 2016) indicated the grouped enrichment of the Notch signaling factors in Volcano Plots (**C**), and the changing frequency of each member was consistent with groups alternations (**D**). **E-F** The Overall survival and Disease/Progression-free estimates with using Kaplan-Meier analysis showed that Notch signaling activation decreased the survival time, and patients tended to bear relapse or resistance in shorter follow-up periods. **G** The relative higher level of TUSC7 indicated longer survival time of all lung carcinoma patients significantly, comparting to the lower expressed groups. Sourced data of Star-Base (v3.0 Project) were collected and analyzed, and the results were calculated with using RPM/Log manner, and a total of 512 lung adenocarcinoma samples were enrolled and applied for analysis. **H** The positive relationship between expression level of miR-146a and level of EGFR was found in total of 512 lung adenocarcinoma samples. Both Notch 1 (**I**) and Notch 2 (**J**) were positively correlated to miR-146a expression, pointing to the oncogenic functions of miR-146a

The important members of Notch signaling [30, 31] were screened for expression patterns with using Pan-Cancer Atlas of TCGA data base. The total samples of 507 patients were collected, and the heat maps indicated the universal overexpression of notch signaling participants (Fig. 1A-B), and EGFR was correlated with aberrant Notch expression. Deep analysis from TCGA (Nat Genet 2016 data base) indicated the grouped enrichment of Notch signaling factors (Fig. 1C), and the changing was consistency in groups harboring most irregulated Notch functions (Fig. 1D).

The overall survival (OS) and Disease/Progression-free survival (DFS/PFS) data were acquired from TCGA data at the CBIOPORTAL FOR CANCER GENOMIC (http://www.cbioportal.org/) [32, 33]. Notch signaling activation decreased the survival time (Fig. 1E), and patients tended to bear relapse or resistance in shorter follow-up periods (Fig. 1F). The overall survival and first progression estimates were analyzed by applying Kaplan-Meier analysis (http://kmplot.com/analysis/) [34–36]. The highly expressed TUSC7 indicated better progression-free estimates in adenocarcinoma, comparing to the lower expressed groups (Fig. 1G). The functional EGFR signaling could transcriptionally activate multiple downstream pathways, and the positive relationship between miR-146a and EGFR was found (Fig. 1H). MiR-146 also correlated with Notch signaling factors expressions (Fig. 1I-J).

### Erlotinib resistant cells harboring notch signaling activation and TUSC7 inhibition

Lung adenocarcinoma cell lines of PC9 and HCC827 were selected for their characteristics of specific EGFR mutant status, and the signatures of PC9 and HCC827 cells referring to erlotinib treatment were shown in Figure S1. The concentration of 0.2 μM Erlotinib was chosen as the function candidate, which decreased the Notch1 (Figure S2A) and Notch2 (Figure S2B) mRNA levels in PC9 and HCC827 cells. The Erlotinib treatment also increased the TUSC7 expression level significantly (Figure S2C), together with EGFR level slightly decreased (Figure S2D). Blotting results showed that the Erlotinib treatment decreased the Notch signaling factors in PC9 and HCC827 cells (Figure S2E).

We constructed Erlotinib resistant lung cancer cells with advancing concentration gradient (Figure S3), and the differentially expressed lncRNAs between Erlotinib sensitive and resistant cells were primarily detected by gene panel selection (Figure S2F). The differentially expressed lncRNAs were pasted for GO analysis of functional identification (Figure S2G). The detailed information showed that TUSC7 of both PC9ER (Figure S2H) and HCC827ER (Figure S2I) decreased greatly, and did not react to Erlotinib treatment, comparing to the Erlotinib sensitive cells. Last but not least Erlotinib failed to inhibit the Notch signaling functions in PC9ER and HCC827ER cells (Figure S2H-I).

### Notch signaling inhibition was required for TUSC7 alleviating of the Erlotinib resistance

**Fig. 2:**
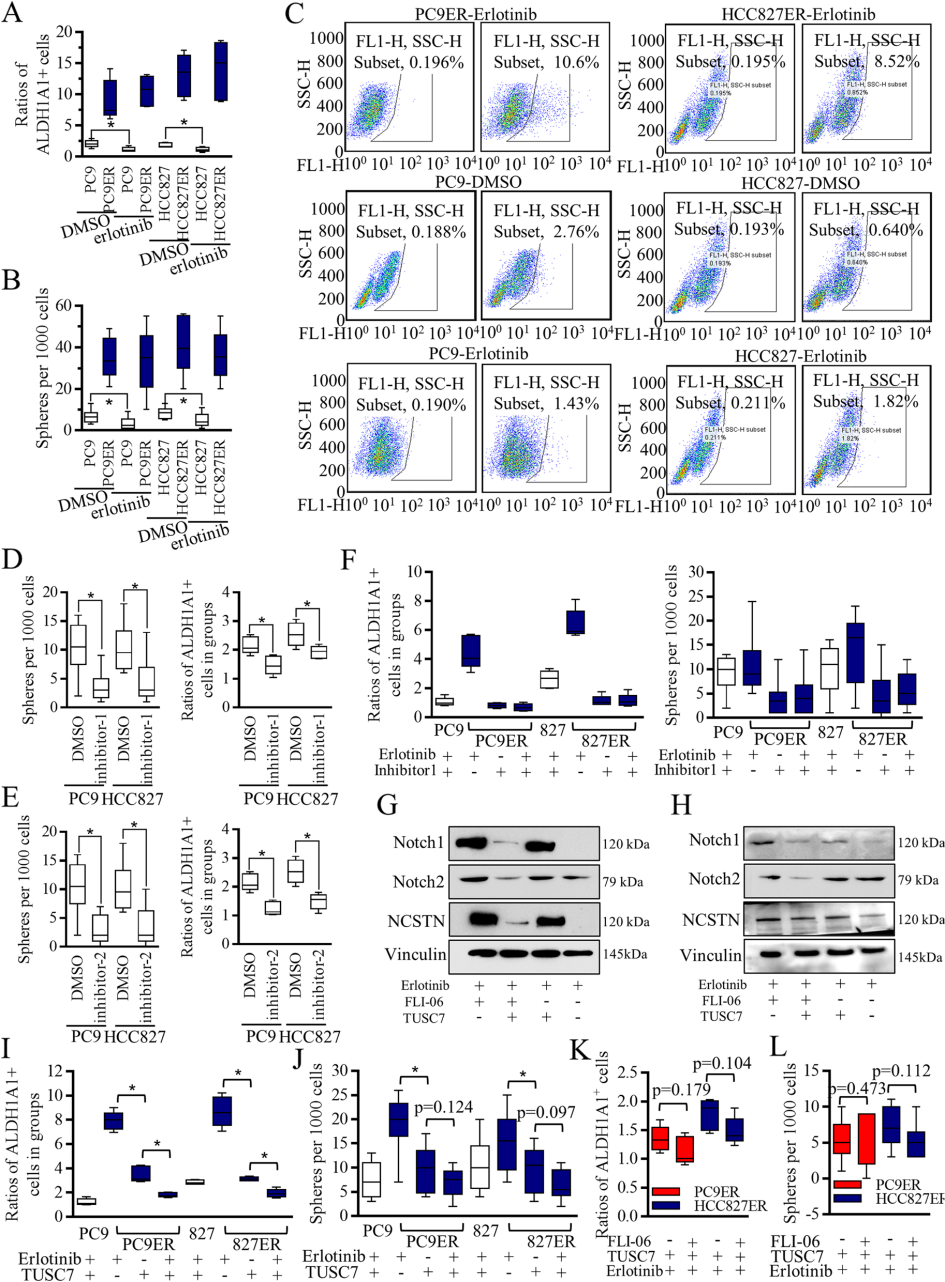
Notch inhibition decreased the self-renewal ability of Erlotinib resistant cells and re-sensitized the resistant cells to Erlotinib. **A** The addition of Erlotinib decreased the ALDH1A1 positive cells of PC9 and HCC827 cells significantly, but did not affect the ratios of Erlotinib resistant PC9ER and HCC827ER cells. **B** The addition of Erlotinib decreased the spheres number of PC9 and HCC827 cells significantly, but did not affect the number of Erlotinib resistant PC9ER and HCC827ER cells. **C** Representative images of ALDEFLUOR isolation were detailed exhibited. Two kinds of Notch signaling inhibitors, FLI-06 (inhibitor-1), and γ-Secretase inhibitor (inhibitor-2) were used. 200 nM of inhibitor-1 (**D**) decreased the self-renewal ability of multiple kinds of lung cancer cells, and 50 nM of inhibitor-2 (**E**) decreased the self-renewal ability of multiple kinds of lung cancer cells. **F** Notch signaling inhibition decreased the stem cells’ ratio of the Erlotinib resistant cells significantly, and further, the much-lowered concentration of Notch signaling inhibitor-1, the 20 nM of FLI-06 sensitized both PC9ER and HCC827ER cells to Erlotinib treatment greatly. Erlotinib alone inhibited the Notch signaling slightly, and lowered concentration of FLI-06 mildly inhibited the Notch signaling, but effectively enhanced the Erlotinib functions in PC9ER (Fig. 3G) and HCC827ER cells (Fig. 3H). Combined TUSC7 and Erlotinib decreased the stem cells ratio greatly in both PC9ER and HCC827ER cells (Fig. 3I-J). **K-L** The stem cells’ renewal suppression evaluation did not show significant differences between TUSC7 alone and the combination of TUSC7 and FLI-06

The stem-like cells were accused for treatment resistance, and to study the stem cells’ renewal ability in contributing to Erlotinib resistance, the ALDH1 phenotype and spheres formation nature were applied. The Erlotinib resistant PC9ER and HCC827ER cells consisted of more stem-like cells (Fig. 2A-B), and Erlotinib treatment failed to decrease the stem cells number (Fig. 2C). Higher stem cells ratio indicated resistant status, and resulted in group resistance. The Notch inhibitors were chosen as FLI-06 (inhibitor-1, ab120633, Abcam), and γ-Secretase inhibitor (inhibitor-2, ab146170, Abcam). Both PC9 and HCC827 cells responded to Notch signaling inhibitors, with stem-like cells ratios decreasing significantly (Fig. 2D-E). The lowered concentration of Notch signaling inhibitors sensitized resistant cells to Erlotinib treatment (Fig. 2F, Figure S4A-B), indicating the synergistic effects of TKI agents and Notch signaling inhibitors.

To study the TUSC7 functions, Lentiviral based TUSC7 expression vesical was introduced into PC9ER and HCC827ER cells. Erlotinib alone inhibited the Notch signaling slightly, but TUSC7 effectively enhanced the Erlotinib functions in PC9ER (Fig. 2G) and HCC827ER cells (Fig. 2H), and stimulated the suppressive functions of Erlotinib in both PC9ER (Figure S4C) and HCC827ER cells (Figure S4D). Moreover, the addition of lowered concentration of Notch signaling inhibitor strengthened TUSC7 functions (Fig. 2G-H).

Next, we found that combined TUSC7 and Erlotinib decreased the stem cells ratio greatly in both PC9ER (Fig. 2I) and HCC827ER cells (Fig. 2J). The stem cells’ renewal suppression evaluation did not show significant differences between TUSC7 alone and the combination of TUSC7 and FLI-06 (Fig. 2K-L, Figure S4E-G). The combined TUSC7 and Erlotinib decreased the stem cells associated signatures, decreasing EMT markers in PC9ER cells (Figure S5A) and HCC827ER cells (Figure S5B).

### TUSC7 sensitization of Erlotinib through miR-146a/notch signaling inhibition was dependent on NUMB restoration

**Fig. 3:**
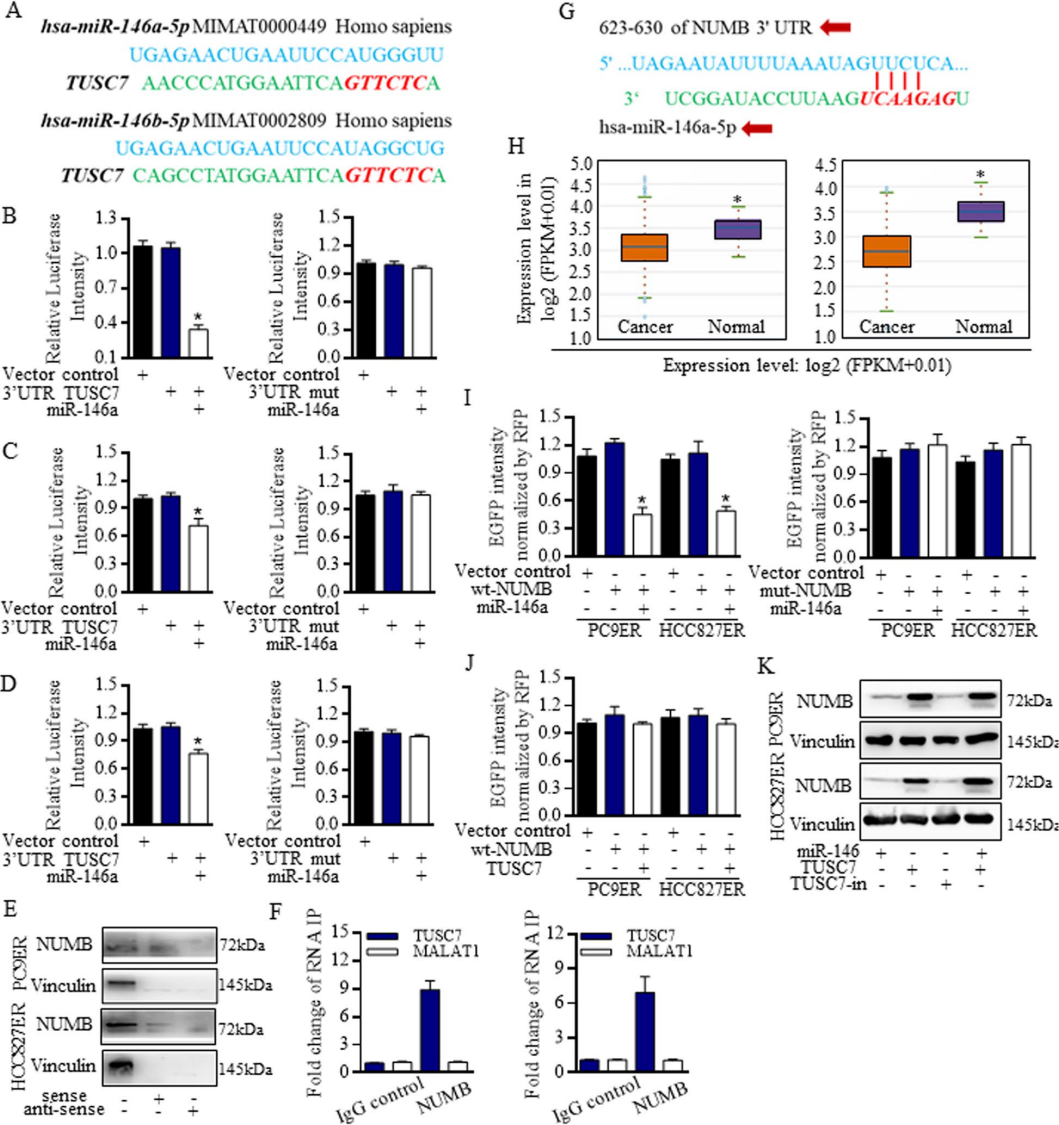
MiR-146a conducted NUMB degradation was blocked by TUSC7 in a sponge combination manner. **A** The predicated connection sites between TUSC7 and its binding partners showed that miR-146a shared the common untranslated regions. **B** Recombined miR-146a mimics decreased the Luc-activity of TUSC7 in 293 T cells. MiR-146a decreased the Luc-activity of TUSC7 in PC9ER cells (**C**) and HCC827ER cells (**D**). **E** Blotting results referring to RNA pull-down test showed the connection between TUSC7 and NUMB in PC9ER and HCC827ER cells. **F** RNA immunoprecipitation revealed that TUSC7 was enriched with NUMB expression in PC9ER cells (Left) and HCC827ER cells (Right), and the IgG was set as the immunoprecipitation control, the MALAT1 was set as the primer control. **G** Informatic screening of the potential miRNAs’ targets suggested that miR-146a may bind to NUMB. **H** The expression level of NUMB with 526 cancer and 59 normal samples in LUAD, and the expressions with 501 cancer and 49 normal samples in LUSC were evaluated, and NUMB decreased greatly in cancer group. **I** The co-transfection of miR-146a mimics and the wide-type reporter plasmid strongly reduced the EGFP intensity, and miR-146a mimics reduced nearly 40% of the TUSC7 luciferase intensity, but not happened in mutant-type reporter plasmid. **J** TUSC7 alone did not reduce the EGFP activity of NUMB. **K** MiR-146a decreased the NUMB expression, which could be rescued by TUSC7, and the TUSC7 inhibition (TUSC7-in) also decreased the NUMB level

To reveal the transduction mechanisms, bioinformatic screening of the possible connections between TUSC7 and its binding partners was conducted. We noticed that miR-146a shared common sequences with the untranslated regions of TUSC7 (Fig. 3A), and miR-146a mimics decreased the Luc-activity of TUSC7 significantly (Fig. 3B) in 293 T cells, which was also confirmed in PC9ER (Fig. 3C) cells and HCC827ER cells (Fig. 3D). To testify the binding probabilities between TUSC7 and its downstream partner, biotin labeled sense and anti-sense RNAs of TUSC7 were used for RNA pull down detection, and the connection between TUSC7 and NUMB in resistant lung adenocarcinoma cells was identified (Fig. 3E). Further, RNA immunoprecipitation revealed that TUSC7 was enriched with NUMB expression in PC9ER cells (Fig. 3F, left) and HCC827ER cells (Fig. 3F right). Informatic screening of the potential miRNAs’ targets suggested that miR-146a may bind to NUMB (Fig. 3G), and NUMB decreased greatly in cancer group (Fig. 3H). The alignment of miR-146a and the 3’UTR of NUMB was constructed through using the enhanced green fluorescent protein (EGFP) reporter assay. The wild-type 3’UTR sequence and the mutant 3’UTR sequence of NUMB were cloned downstream from the EGFP-coding sequence respectively, to construct the reporter plasmid and the mutant vector. The co-transfection of miR-146a mimics and the wide-type reporter plasmid strongly reduced the EGFP intensity (Fig. 3I, left), but not happened in mutant-type reporter plasmid (Fig. 3I, right). On the contrary, TUSC7 did not reduce the EGFP activity of the NUMB (Fig. 3J). MiR-146a decreased the NUMB expression, which could be rescued by TUSC7, and the TUSC7 inhibition (TUSC7-in) also decreased the NUMB at the protein level (Fig. 3K).

### m6A in resistant cells contributed to TUSC7 inhibition and miR-146a overexpression

**Fig. 4:**
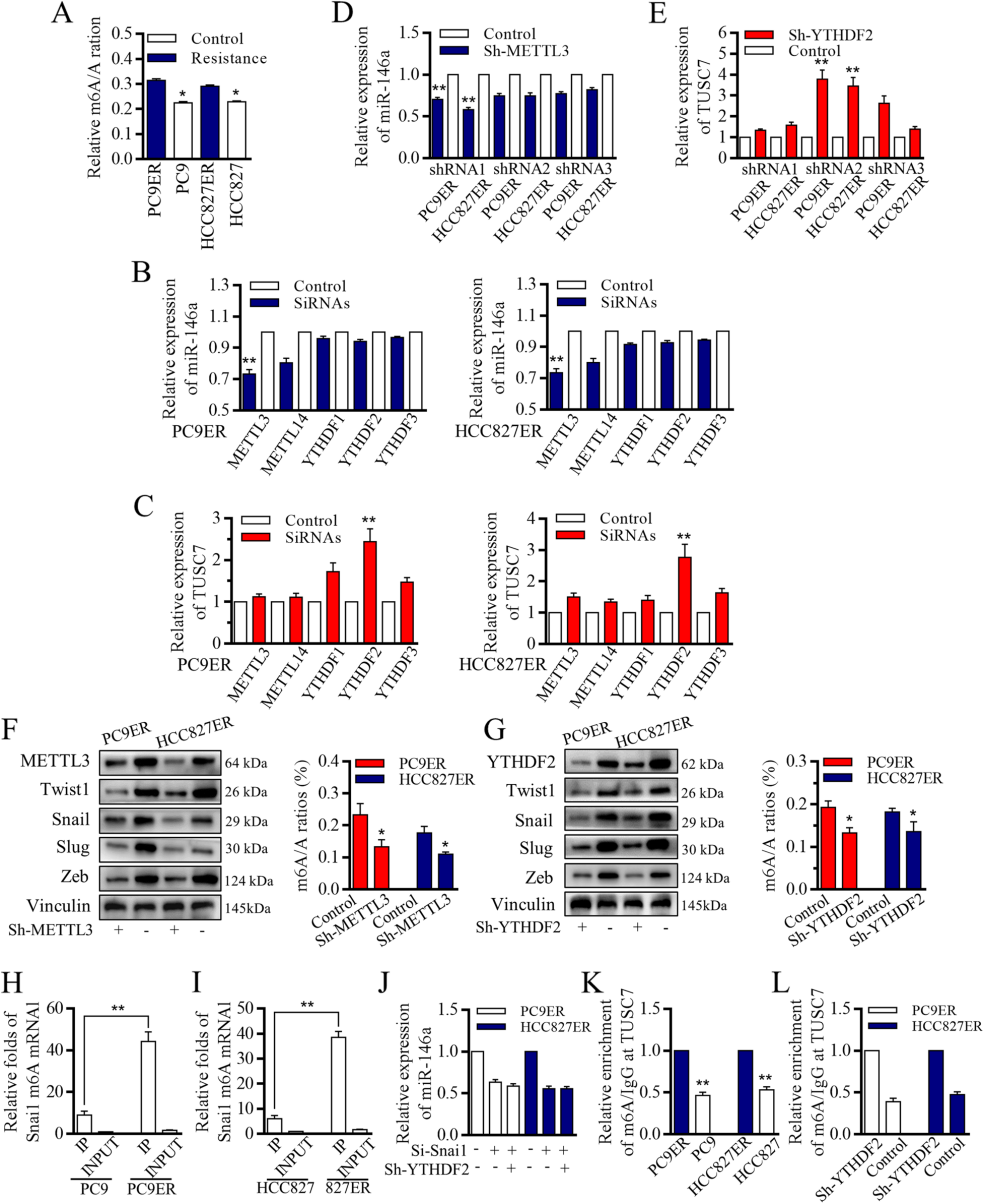
m6A status was associated with TUSC7 inhibition and snail relating miR-146a overexpression. **A** M6A levels of RNAs from resistant cells were statistically more abundant than sensitive original cells. METTL3 affected the miR-146a level (**B**), and YTHDF2 affected TUSC7 level (**C**). **D-E** The results were all confirmed by using the lentiviral based METTL3/YTHDF2 knock-down systems. **F-G** Dysregulated METTL3 and YTHDF2 affected the m6A, and then determined different EMT and stemness feature in resistant PC9ER cells and HCC827ER cells. **H-I** METTL3 inhibition decreased m6A at Snai1. **J** Snai1 inhibition failed to activate the miR-146a promoter activity. **K** The m6A at TUSC7 level increased in resistant cells, and the recognition of TUSC7 m6A peak by YTHDF2 degraded and downregulated the TUSC7 expression. **L** The Me-RIP assay confirmed that the high abundance of m6A modification in cells with YTHDF2 inhibition

The pluripotency status of resistant PC9ER and HCC827ER cells contributed to specific miR-146a and TUSC7 patterns, and to characterize the roles of m6A in therapy resistance, we investigated the variations of m6A levels, and identified that the m6A levels of total RNAs from resistant cells were statistically more abundant than sensitive original cells by using LC/MS (Fig. 4A). To further characterize the roles of m6A in generating the resistance, we used siRNAs to tentatively test the m6A related processers in controlling of TUSC7 and miR-146a. METTL3 affected the miR-146a level (Fig. 4B), and YTHDF2 affected TUSC7 level (Fig. 4C),and results were all confirmed by using the lentiviral based METTL3/YTHDF2 knock-down systems (Fig. 4D-E). Dysregulated METTL3 (Fig. 4F) and YTHDF2 (Fig. 4G) affected the EMT and pluripotency features through controlling m6A status. Moreover, decreasing either METTL3 or YTHDF2 significantly altered Snail and EMT factors expressions. m6A at Snai1 mRNA was greatly increased in resistant cells [26, 37], and we found the METTL3 inhibition decreased m6A at Snai1(Fig. 4H-I), which later failed to activate the miR-146a promoter (Fig. 4J) [38]. In the other hand, the m6A at TUSC7 level increased in resistant cells (Fig. 4K), and the recognition of TUSC7 m6A peak by YTHDF2 degraded TUSC7 [39, 40]. The Me-RIP (methylated RNA immunoprecipitation) assay confirmed that the high abundance of m6A modification in cells with YTHDF2 inhibition (Fig. 4L). All the results revealed that m6A determined the expression levels of miR-146a and TUSC7 in resistant cells, sustaining the balanced status.

### TUSC7 formed feedback loop with miR-146/notch signaling and lead the Erlotinib re-sensitization

**Fig. 5:**
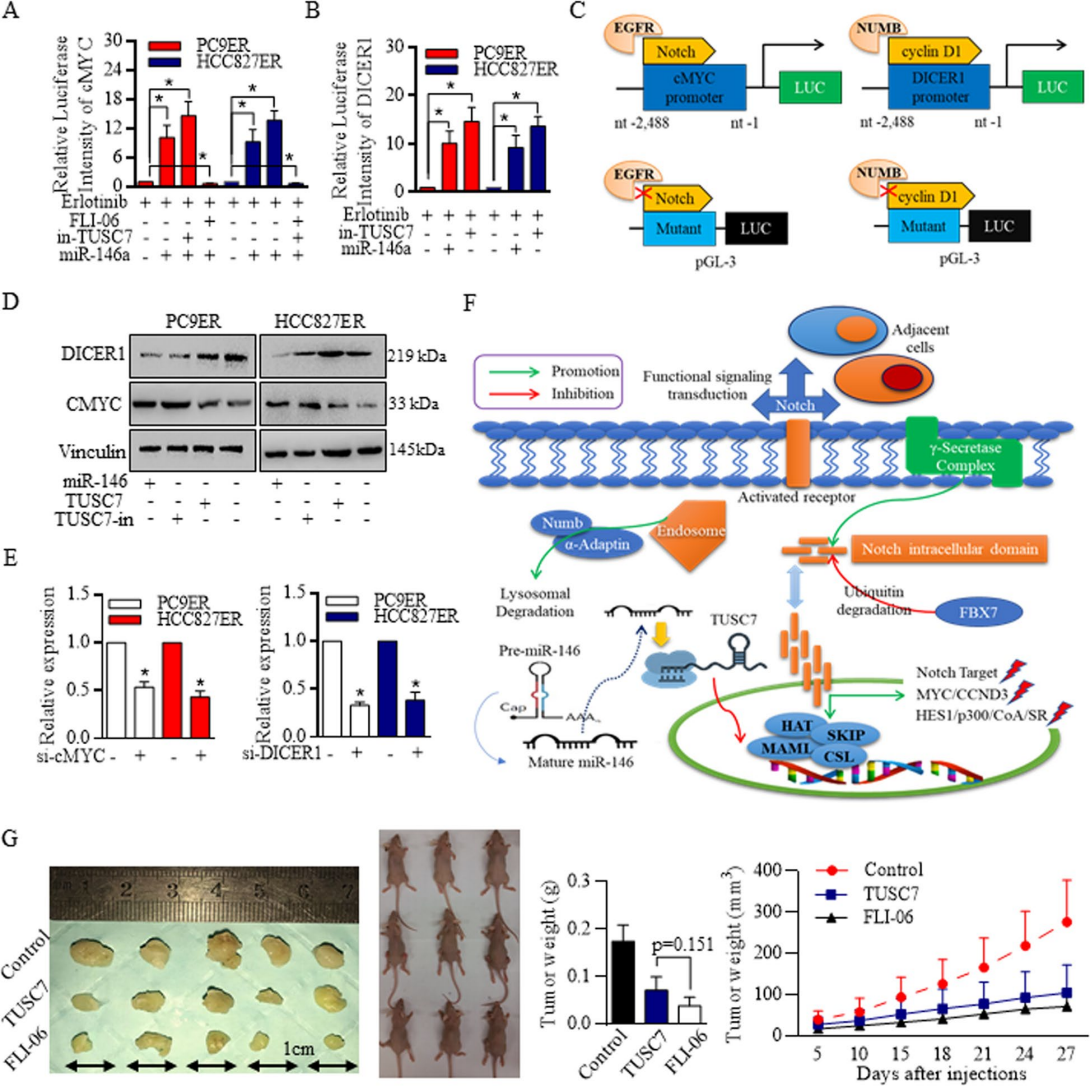
TUSC7 sensitized Erlotinib treatment and formed feedback loop with miR-146/Notch signaling cascade. **A** Notch signaling inhibition resulted in cMYC promoter activity decreasing, and TUSC7 inhibition strongly increased the activity. **B** Inhibition of TUSC7 increased miR-146a expression, and then stimulated the DICER1 activity. **C** The scheme image represented the cMYC promoter detection modes. **D** Western blotting confirmed that in PC9ER and HCC827ER cells, miR-146 and TUSC7 acted the opposite way to promote the DICER1/CCND1 expression. **E** TUSC7 and miR-146a was sustained by cMYC (Left) and DICER1 (Right) respectively. **F** The regulative signaling pathways were drafted and illustrated for detailed exhibition. **G** In vivo study confirmed the effective inhibition of TUSC7 exhibited on tumor growth, and the Notch signaling inactivation by using FLI-06 also suppressed the in vivo tumor expansion

The unique m6A at different RNA sequences determined intrinsic RNA expressions, cellular homeostasis may be disturbed when manipulating certain lncRNA candidate. CMYC was proved previously to promote the translation of lncRNAs [41–43], and DICER1 was critical for miRNAs maturation [44–46]. Results showed that in PC9ER and HCC827ER cells, Notch signaling inhibition lead to cMYC promoter activity decreasing, and TUSC7 inhibition strongly increased the activity (Fig. 5A). Inhibition of TUSC7 increased miR-146a expression, and then stimulated the DICER1 activity (Fig. 5B).

The scheme image represented the cMYC promoter detection modes (Fig. 5C), and the mutation blocked the EGFR/Notch signaling feedback loop on lncRNA transcription induction. Western blotting further confirmed that in PC9ER and HCC827ER cells, miR-146 and TUSC7 acted the opposite way to promote the DICER1/CCND1 expression (Fig. 5D). TUSC7 and miR-146a was sustained by cMYC (Fig. 5E, left) and DICER1 (Fig. 5E, right) respectively. The regulative signaling pathways were drafted and illustrated for detailed exhibition (Fig. 5F), the dysregulated TUSC7 and miR-146a formed the feedback loop with their downstream effectors to sustain the new homeostasis.

In vivo study confirmed the effective inhibition of TUSC7 exhibited on tumor growth, and the Notch signaling inactivation by using FLI-06 also suppressed the in vivo tumor expansion (Fig. 5G), further proved its suppressive functions.

## Conclusion

Lung cancer treatments have been refined greatly, with constantly and novelly emerging components, and the small molecular compounds improved the life quality and expected response to therapies in patients carrying sensitive EGFR mutants [47–49]. Surgery manners have been evolved continuously with little operation wound and duration, however, the improvement has brought little progress on overall survival [50–52]. The 1st generation of small tyrosine kinase inhibitors subversively altered the lung cancer treatments, and the “small step” has made the “major progress”, increasing the PFS with little suffering.

Reality is cruel. No matter how ideal results the TKIs treatment has brought, patients with advanced lung cancer will inevitably face the therapy resistance and disease recurrence [53–55]. To improve the TKIs sensitization and to overcome resistance will be the headline goal in the current emergency [56]. Many factors were thought to be correlated with 1st generation of TKI functions’ achieving, and certain genes and compounds sensitized the treatments by oncogenic signaling repression, but detailed mechanistic regulations have not been illustrated clearly ever [10, 57].

We have been focusing on noncoding RNAs related cancer emergence, progression, stemness features and therapy response for decades, and have revealed some important factors that may contribute to [44, 45, 58]. Previously, we identified the miR-146 functions in lung adenocarcinoma with suppressive affections on stem cells’ renewal, and the tentative exploration of TKIs treatment improvements will facilitate its practical application.

In this study, we identified the inhibitive roles of TUSC7 in lung cancer progression, and after acquiring the Erlotinib resistant cells, gene panel was used for massive assessing of the dysregulated non-coding RNAs. We first explored the features of candidate genes in association with resistance, and the activated miR-146a/Notch signaling was sustained in resistant stem cells in a m6A dependent manner of and METTL3/Snail cascade. M6A related YTHDF2 mediation of suppressed TUSC7 also contributed to resistant features. Functionally, m6A controlled the stemness of EMT features through METTL3 and YTHDF2 in resistant PC9ER and HCC827ER cells.

In detail, TUSC7 sensitized the Erlotinib effects, and decreased the stem cells ratio through Notch signaling inhibition. Bench study showed that, TUSC7 sponged to miR-146 and then released NUMB to control Notch signaling, the latter of which was critical for maintaining cancer stem cells (CSCs) pool. DICER1 and cMYC activity was critical for sustaining the non-coding RNAs maturation [41, 43, 46, 59, 60], the sponge style of TUSC7 regulation toward to miR-146 released the controlling of NUMB expression in PC9ER and HCC827ER cells, which manipulated the DICER and cMYC cascade inner cytoplasm [43, 46, 61, 62]. The absence of either cMYC or DICER1 will lead to TUSC7 and miR-146 decreasing respectively, formed the closed circle to maintain the new balance.

To be concluded, the lncRNA of TUSC7 affected the cancer progression and stem cells renewal, and TUSC7 suppression of Notch signaling determines the Erlotinib treatment response. PC9ER and HCC827ER cells harbors much more stem-like groups, which dominated in therapy response, and their resistance could be reversed by Notch signaling inactivation. Interestingly and importantly, the intrinsic miR-146 and TUSC7 levels are monitored and sustained by m6A effectors, and disturbing the miR-146 and TUSC7 expression patterns will push themselves to form the circling loop to sustain the new homeostasis. Further in clinics, the combined using of TKIs and Notch specific inhibitory non-coding RNAs will pave the way for yielding the susceptibility to targeted therapy in lung cancer.

## Supplementary Information

**Additional file 1: Supplemental Figure 1.** The sensitivity of Erlotinib in treating Lung adenocarcinoma harboring mutant EGFR. The sensitivity analysis was carried with using shared data of Genomics of Drug Sensitivity in Cancer at the SANGER site. A. The Erlotinib sensitivity referring to lung cancer samples of PAN data were rankly exhibited. B. The Erlotinib sensitivity referring to lung adenocarcinoma samples were rankly exhibited. Both PC9 (C) and HCC827 (D) were sensitive to Erlotinib treatment with concentration much lower than IC50. E. EGFR mutant lung cancer cells are very sensitive to Erlotinib treatment, comparing to that of lung cancer cells with wild type EGFR.

**Additional file 2: Supplemental Figure 2.** Signatures of the Erlotinib resistant lung adenocarcinoma cells. A. Erlotinib decreased the Notch1 mRNA level in both PC9 and HCC827 cells. B. Erlotinib decreased the Notch2 mRNA level in both PC9 and HCC827 cells. C. Erlotinib increased the TUSC7 expression level significantly in both PC9 and HCC827 cells. D. Addition of Erlotinib did not change the EGFR expression level in both PC9 and HCC827 cells. E. Erlotinib decreased the Notch signaling factors in PC9 and HCC827 cells. F. The newly established PC9ER and HCC827ER were analyzed for lncRNAs expression patterns, and the primarily results were showed in Heatmap. G. The differentially expressed LncRNAs between Erlotinib resistant cells and sensitive cells were categorized by using GO analysis, and TUSC7 was supposed to be inhibited in PC9ER and HCC827ER cells. Real-time PCR further confirmed the suppression of TUSC7 in PC9ER (H) and HCC827ER (I) cells.

**Additional file 3: Supplemental Figure 3.** Establishing the Erlotinib resistant cells. A. Osimertinib was dissolved in dimethyl sulfoxide (DMSO), and a total of 1 × 10^6^ cells/ml of H1975 cells were seeded in a 6-wells plate and incubated in RPMI-1640 medium containing Osimertinib. The initial concentrations of Osimertinib were started with a concentration equal to the half-maximal inhibitory concentration of H1975 cells. After a cycle of Osimertinib treatment, only a small percentage of cells remained. Once cells had resumed normal growth and returned to 80% confluence under the light microscope, the next cycle began. The drug concentration was gradually increased for the next cycle until cells could survive with 10 μM Osimertinib. After 6 months, the H1975OR cells were successfully established and were then harvested for RNAs analysis. B. The images of gene probes detection were exhibited for illustration.

**Additional file 4: Supplemental Figure 4.** TUSC7 re-sensitized the resistant PC9ER cells and HCC827ER cells through Notch signaling inhibition. A-B. All lung cancer cells responded to Notch signaling inhibitors greatly, and the much-lowered concentration of Notch signaling inhibitor sensitized both PC9ER and HCC827ER cells to Erlotinib treatment. TUSC7 stimulated the suppressive functions of Erlotinib in both PC9ER (C) and HCC827ER cells (D). E. Differences of the proliferation inhibition ratios between TUSC7 alone and the combination of TUSC7 and FLI-06 was insignificant. F-G. Supplemented images.

**Additional file 5: Supplemental Figure 5.** Blotting results to prove that TUSC7 functioned through Notch signaling inhibition in resistant PC9ER cells and HCC827ER cells. The combined TUSC7 and Notch signaling inhibitor decreased the stem cells associated signatures, and decreased the EMT markers in PC9ER cells (A) and HCC827ER cells (B), but the inhibitory result was similar to that of using either TUSC7 or Notch signaling inhibitor alone.

## Data Availability

The datasets during and/or analyzed during the current study available from the corresponding author on reasonable request.

## Abbreviations

TKIs: Tyrosin Kinase related Inhibitors
lncRNAs: long non-coding RNAs
circRNAs: circular RNAs
m6A: N-6-methyladenosine
RT-qPCR: Real-time quantitative PCR
ALDH: Aldehyde dehydrogenase
BAAA: Bodipy-aminoacetaldehyde
DEAB: Diethylaminobenzaldehyde
EGFP: Green fluorescent protein
Me-RIP: Methylated RNA immunoprecipitation
