# Correction: M6A associated TSUC7 inhibition contributed to Erlotinib resistance in lung adenocarcinoma through a notch signaling activation dependent way

**DOI:** 10.1186/s13046-023-02760-8

**Published:** 2023-07-24

**Authors:** Kai Li, Zi-Yang Peng, Shan Gao, Qing-Shi Wang, Rui Wang, Xiang Li, Guo-Dong Xiao, Jing Zhang, Hong Ren, Shou-Ching Tang, Xin Sun

**Affiliations:** 1 Department of Thoracic Surgery, the Second Department of Thoracic Surgery, Department of Thoracic Surgery and Oncology, Cancer Center, the First Afliated Hospital of Xi’an Jiaotong University, 277 Yanta West Road, Xi’an City, 710061 Shaanxi Province China; 2 Department of Pathology, Anatomy & Cell Biology, Sidney Kimmel Cancer Center, Thomas Jeferson University, Philadelphia, PA 19107 USA; 3 Oncology Department, the First Afliated Hospital of Zhengzhou University, Zheng Zhou City, 450052 Henan Province China; 4 University of Mississippi Medical Center, Cancer Center and Research Institute, 2500 North State Street, Jackson, MS 39216 USA

**Correction:** ***J Exp Clin Cancer Res*** **40, 325 (2021)**

**https://doi.org/10.1186/s13046-021-02137-9**

Following publication of the original article [1], an error was identified Figs. 2 and 4. The original alternative image was mistakenly used in Fig. 2H, while in the editing of Fig. 4G, one Western blot band was not carefully discerned and was duplicated.

The correct figures are presented below:

**Fig. 2:**
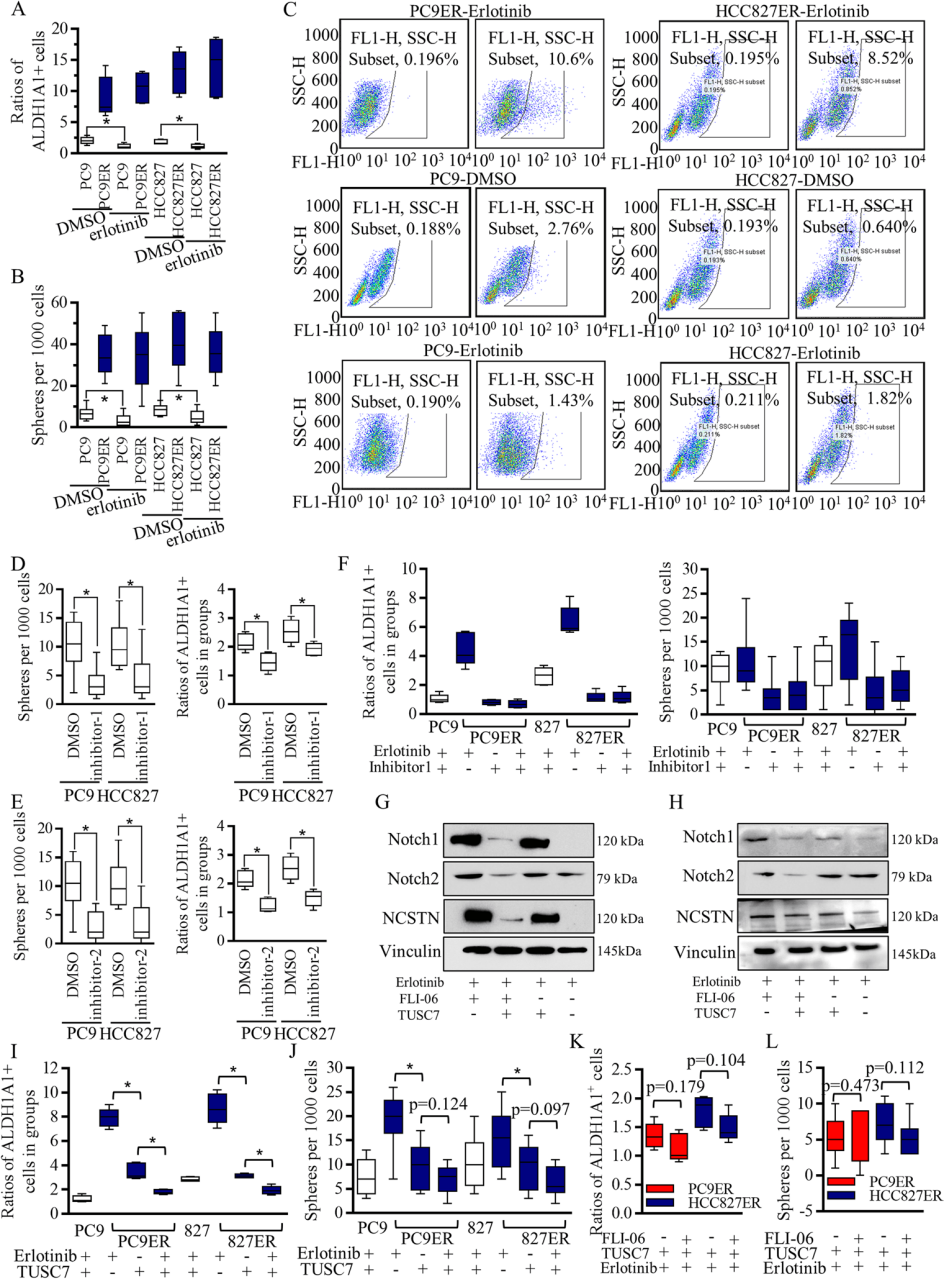
Notch inhibition decreased the self-renewal ability of Erlotinib resistant cells and re-sensitized the resistant cells to Erlotinib. **A** The addition of Erlotinib decreased the ALDH1A1 positive cells of PC9 and HCC827 cells significantly, but did not affect the ratios of Erlotinib resistant PC9ER and HCC827ER cells. **B** The addition of Erlotinib decreased the spheres number of PC9 and HCC827 cells significantly, but did not affect the number of Erlotinib resistant PC9ER and HCC827ER cells. **C** Representative images of ALDEFLUOR isolation were detailed exhibited. Two kinds of Notch signaling inhibitors, FLI-06 (inhibitor-1), and γ-Secretase inhibitor (inhibitor-2) were used. 200 nM of inhibitor-1 (**D**) decreased the self-renewal ability of multiple kinds of lung cancer cells, and 50 nM of inhibitor-2 (**E**) decreased the self-renewal ability of multiple kinds of lung cancer cells. **F** Notch signaling inhibition decreased the stem cells’ ratio of the Erlotinib resistant cells significantly, and further, the much-lowered concentration of Notch signaling inhibitor-1, the 20 nM of FLI-06 sensitized both PC9ER and HCC827ER cells to Erlotinib treatment greatly. Erlotinib alone inhibited the Notch signaling slightly, and lowered concentration of FLI-06 mildly inhibited the Notch signaling, but effectively enhanced the Erlotinib functions in PC9ER (Fig. 3G) and HCC827ER cells (Fig. 3H). Combined TUSC7 and Erlotinib decreased the stem cells ratio greatly in both PC9ER and HCC827ER cells (Fig. 3I-J). **K-L** The stem cells’ renewal suppression evaluation did not show significant differences between TUSC7 alone and the combination of TUSC7 and FLI-06

**Fig. 4:**
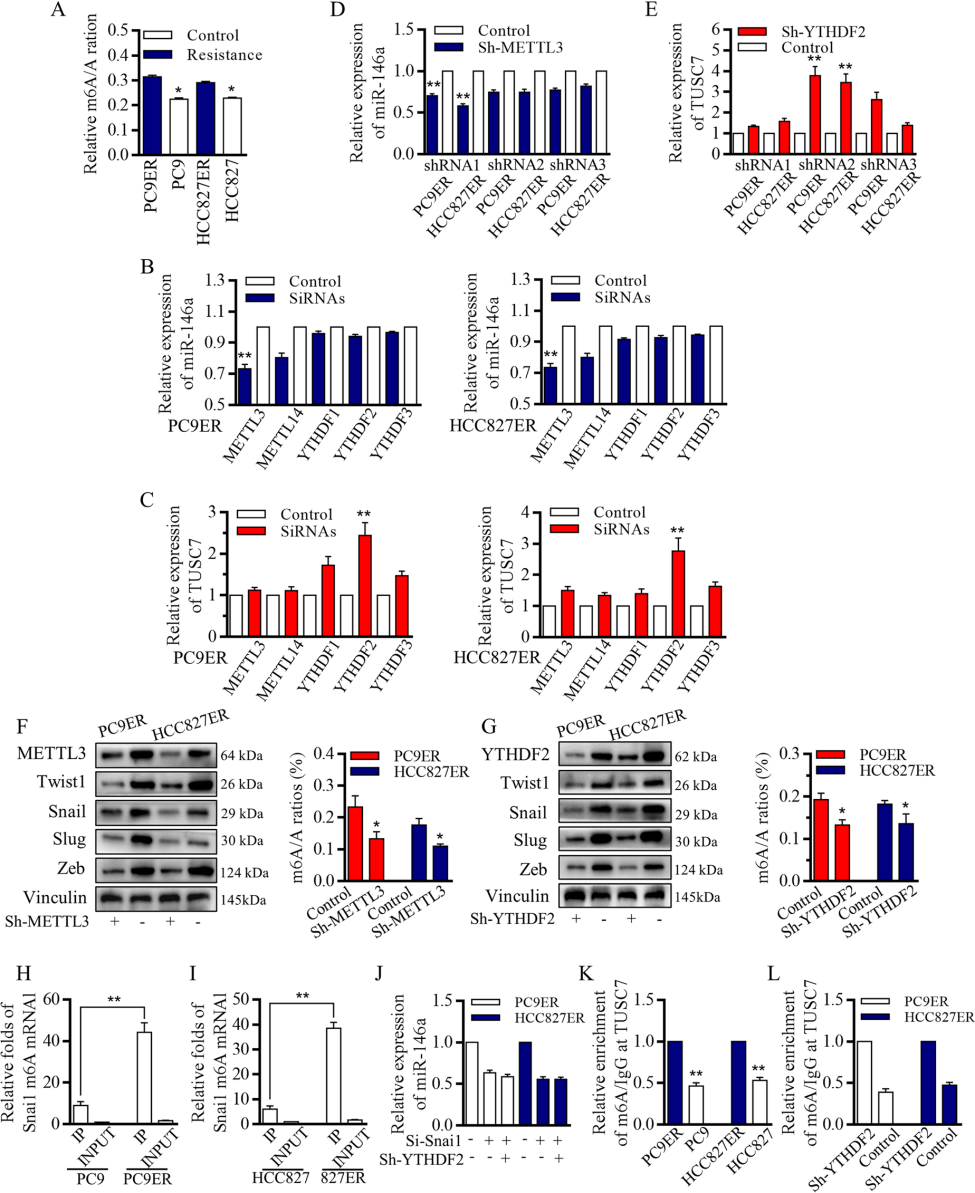
m6A status was associated with TUSC7 inhibition and snail relating miR-146a overexpression. **A** M6A levels of RNAs from resistant cells were statistically more abundant than sensitive original cells. METTL3 affected the miR-146a level (**B**), and YTHDF2 affected TUSC7 level (**C**). **D-E** The results were all confirmed by using the lentiviral based METTL3/YTHDF2 knock-down systems. **F-G** Dysregulated METTL3 and YTHDF2 affected the m6A, and then determined different EMT and stemness feature in resistant PC9ER cells and HCC827ER cells. **H-I** METTL3 inhibition decreased m6A at Snai1. **J** Snai1 inhibition failed to activate the miR-146a promoter activity. **K** The m6A at TUSC7 level increased in resistant cells, and the recognition of TUSC7 m6A peak by YTHDF2 degraded and downregulated the TUSC7 expression. **L** The Me-RIP assay confirmed that the high abundance of m6A modification in cells with YTHDF2 inhibition.

The correction does not affect the overall Conclusion of the article. The original article has been corrected.
