# Supplementary figures and images for: M6A associated TSUC7 inhibition contributed to Erlotinib resistance in lung adenocarcinoma through a notch signaling activation dependent way
Source: J Exp Clin Cancer Res. 2021 Oct 16;40:325. doi: 10.1186/s13046-021-02137-9 (PMC8520306; doi:10.1186/s13046-021-02137-9)

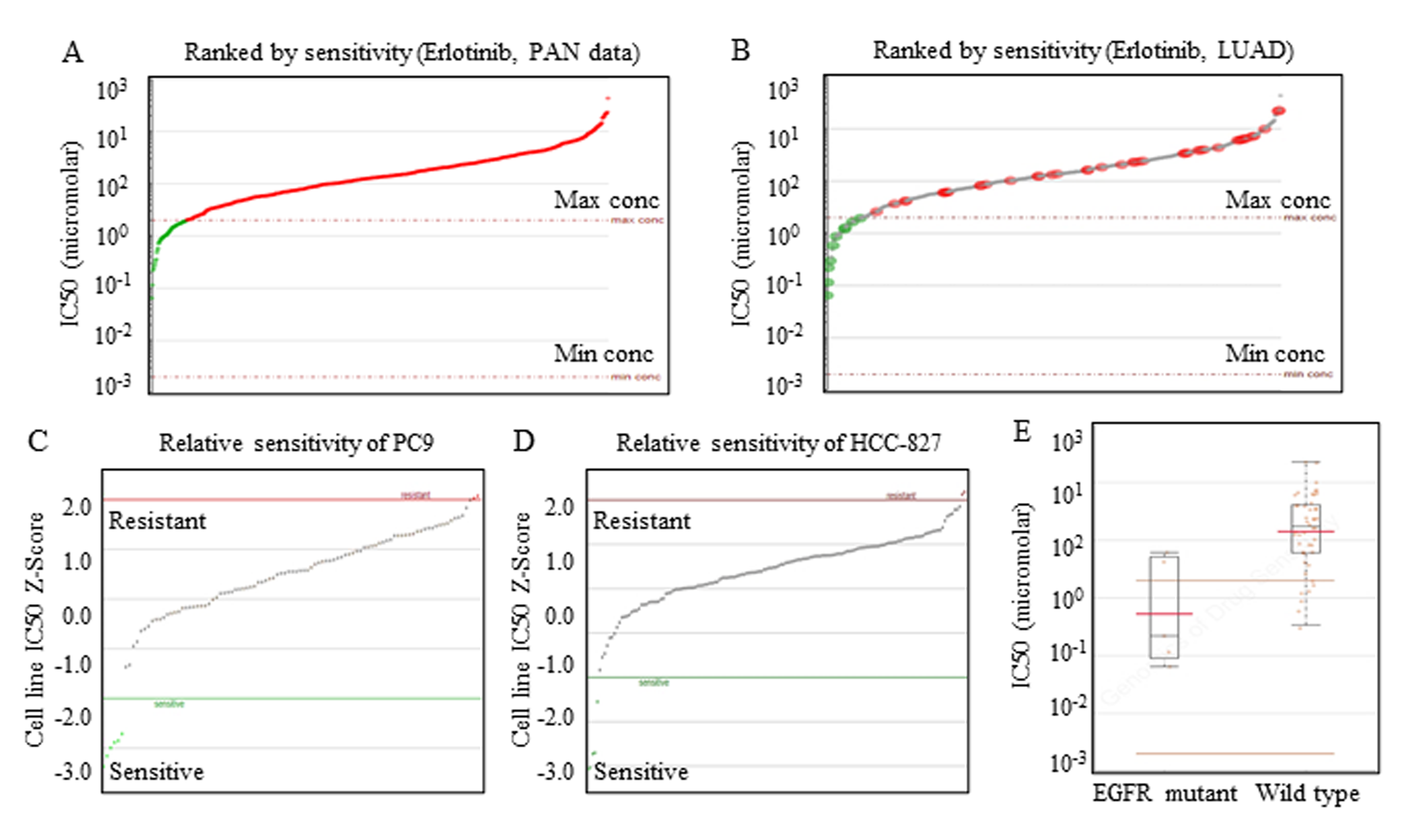

Supplement: Supplementary file 1 — Additional file 1: Supplemental Figure 1. The sensitivity of Erlotinib in treating Lung adenocarcinoma harboring mutant EGFR. The sensitivity analysis was carried with using shared data of Genomics of Drug Sensitivity in Cancer at the SANGER site. A. The Erlotinib sensitivity referring to lung cancer samples of PAN data were rankly exhibited. B. The Erlotinib sensitivity referring to lung adenocarcinoma samples were rankly exhibited. Both PC9 (C) and HCC827 (D) were sensitive to Erlotinib treatment with concentration much lower than IC50. E. EGFR mutant lung cancer cells are very sensitive to Erlotinib treatment, comparing to that of lung cancer cells with wild type EGFR. [file 13046_2021_2137_MOESM1_ESM.tif]

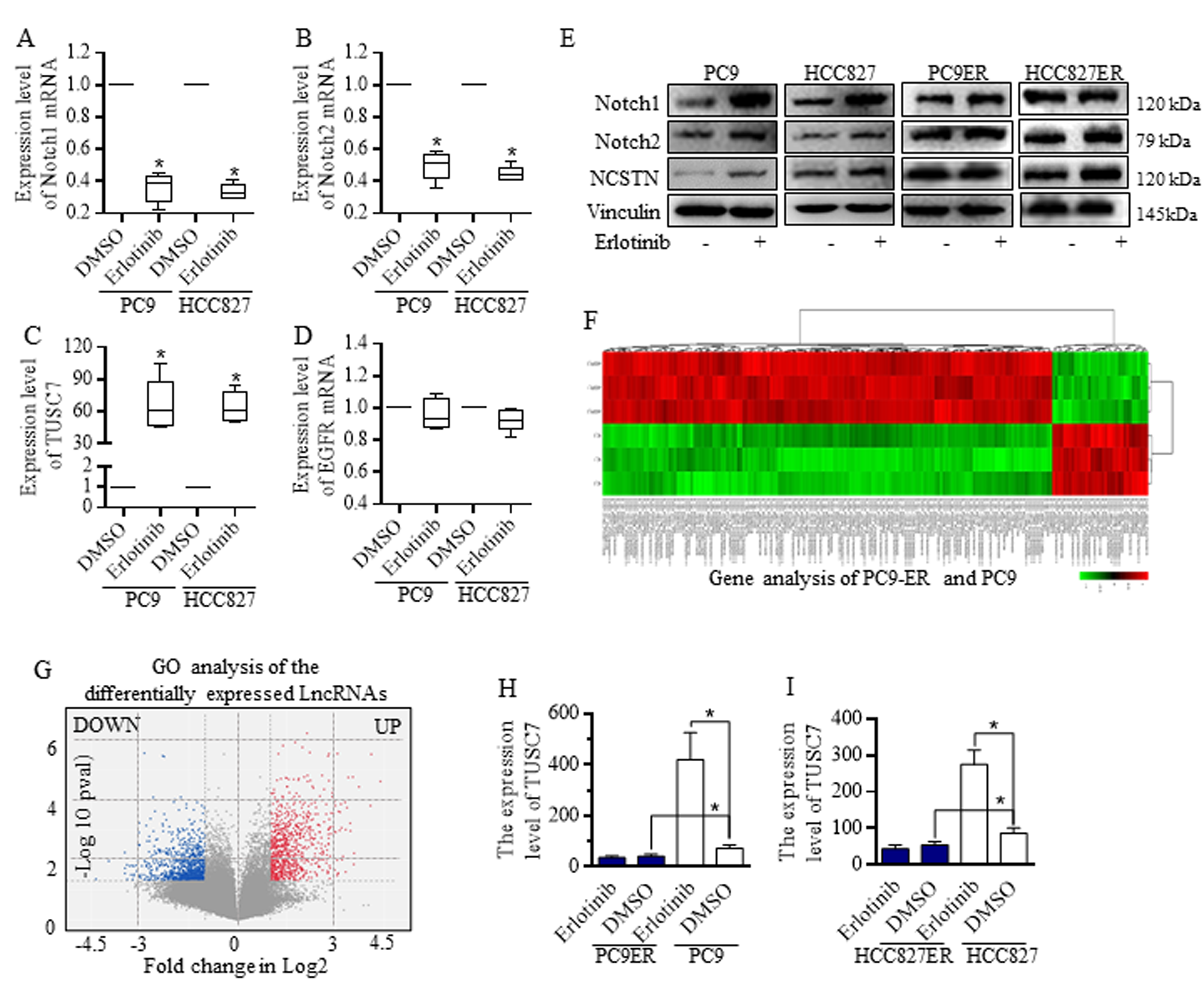

Supplement: Supplementary file 2 — Additional file 2: Supplemental Figure 2. Signatures of the Erlotinib resistant lung adenocarcinoma cells. A. Erlotinib decreased the Notch1 mRNA level in both PC9 and HCC827 cells. B. Erlotinib decreased the Notch2 mRNA level in both PC9 and HCC827 cells. C. Erlotinib increased the TUSC7 expression level significantly in both PC9 and HCC827 cells. D. Addition of Erlotinib did not change the EGFR expression level in both PC9 and HCC827 cells. E. Erlotinib decreased the Notch signaling factors in PC9 and HCC827 cells. F. The newly established PC9ER and HCC827ER were analyzed for lncRNAs expression patterns, and the primarily results were showed in Heatmap. G. The differentially expressed LncRNAs between Erlotinib resistant cells and sensitive cells were categorized by using GO analysis, and TUSC7 was supposed to be inhibited in PC9ER and HCC827ER cells. Real-time PCR further confirmed the suppression of TUSC7 in PC9ER (H) and HCC827ER (I) cells. [file 13046_2021_2137_MOESM2_ESM.tif]

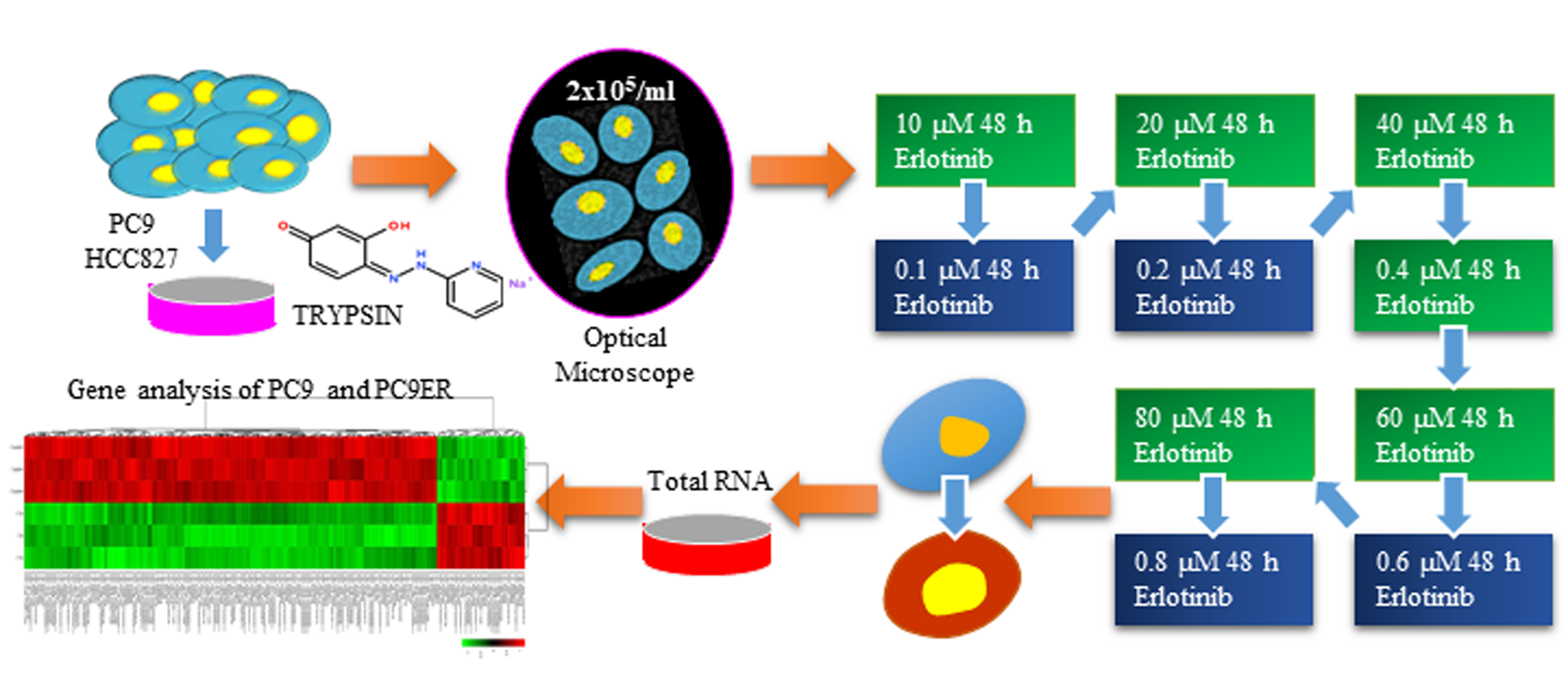

Supplement: Supplementary file 3 — Additional file 3: Supplemental Figure 3. Establishing the Erlotinib resistant cells. A. Osimertinib was dissolved in dimethyl sulfoxide (DMSO), and a total of 1 × 10^6^ cells/ml of H1975 cells were seeded in a 6-wells plate and incubated in RPMI-1640 medium containing Osimertinib. The initial concentrations of Osimertinib were started with a concentration equal to the half-maximal inhibitory concentration of H1975 cells. After a cycle of Osimertinib treatment, only a small percentage of cells remained. Once cells had resumed normal growth and returned to 80% confluence under the light microscope, the next cycle began. The drug concentration was gradually increased for the next cycle until cells could survive with 10 μM Osimertinib. After 6 months, the H1975OR cells were successfully established and were then harvested for RNAs analysis. B. The images of gene probes detection were exhibited for illustration. [file 13046_2021_2137_MOESM3_ESM.tif]

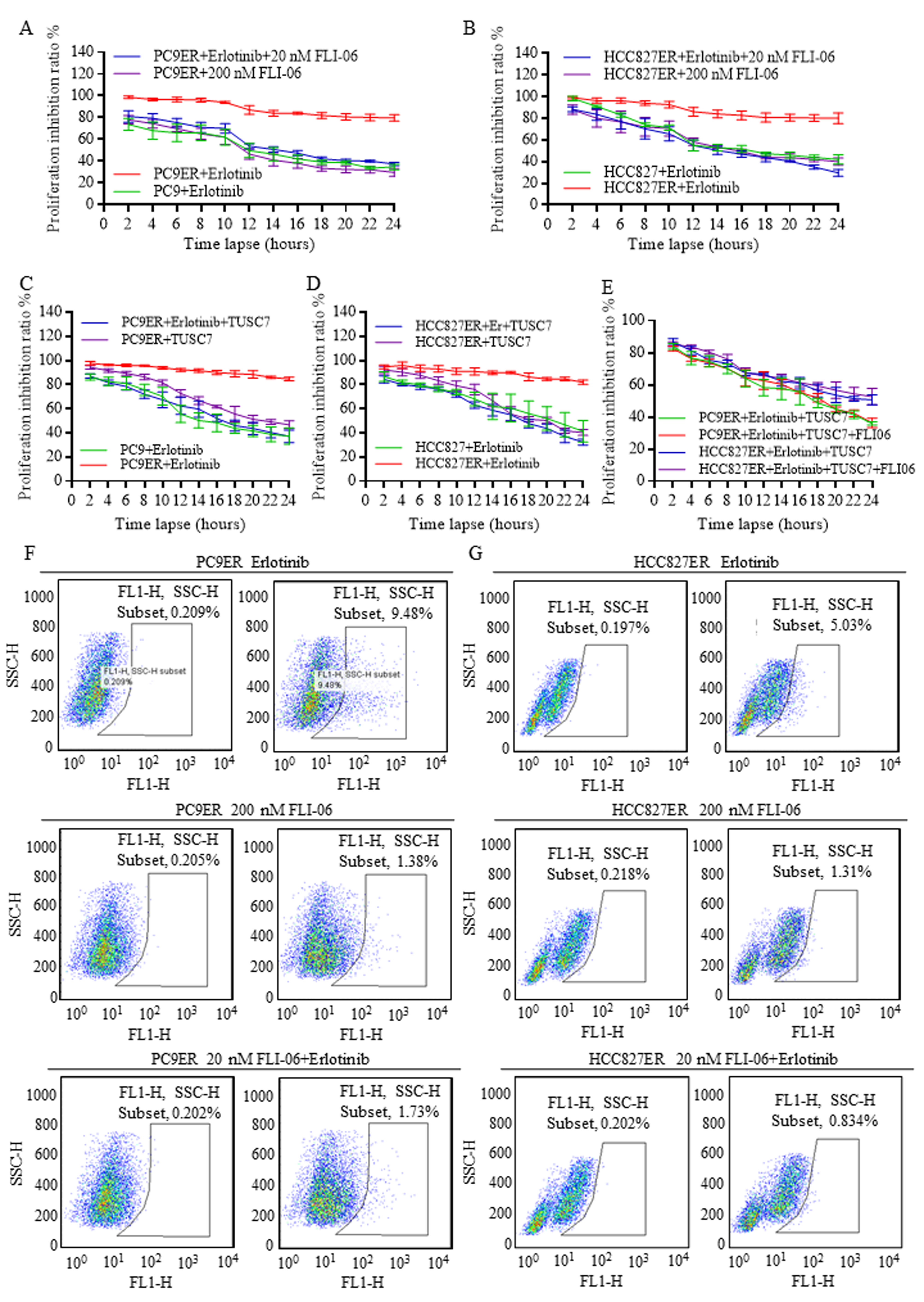

Supplement: Supplementary file 4 — Additional file 4: Supplemental Figure 4. TUSC7 re-sensitized the resistant PC9ER cells and HCC827ER cells through Notch signaling inhibition. A-B. All lung cancer cells responded to Notch signaling inhibitors greatly, and the much-lowered concentration of Notch signaling inhibitor sensitized both PC9ER and HCC827ER cells to Erlotinib treatment. TUSC7 stimulated the suppressive functions of Erlotinib in both PC9ER (C) and HCC827ER cells (D). E. Differences of the proliferation inhibition ratios between TUSC7 alone and the combination of TUSC7 and FLI-06 was insignificant. F-G. Supplemented images. [file 13046_2021_2137_MOESM4_ESM.tif]

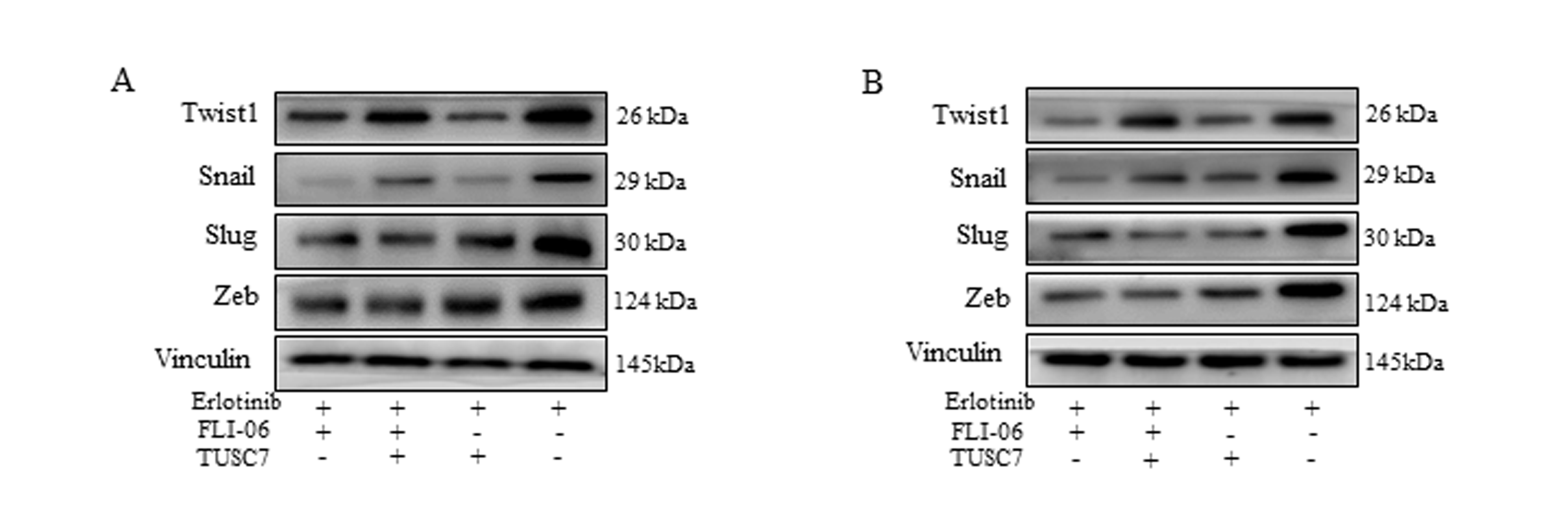

Supplement: Supplementary file 5 — Additional file 5: Supplemental Figure 5. Blotting results to prove that TUSC7 functioned through Notch signaling inhibition in resistant PC9ER cells and HCC827ER cells. The combined TUSC7 and Notch signaling inhibitor decreased the stem cells associated signatures, and decreased the EMT markers in PC9ER cells (A) and HCC827ER cells (B), but the inhibitory result was similar to that of using either TUSC7 or Notch signaling inhibitor alone. [file 13046_2021_2137_MOESM5_ESM.tif]
